# Are Distal and Proximal Visual Cues Equally Important during Spatial Learning in Mice? A Pilot Study of Overshadowing in the Spatial Domain

**DOI:** 10.3389/fnbeh.2017.00109

**Published:** 2017-06-06

**Authors:** Marie Hébert, Jan Bulla, Denis Vivien, Véronique Agin

**Affiliations:** ^1^Normandie Université, UNICAEN, INSERM, Physiopathology and Imaging of Neurological DisordersCaen, France; ^2^Center for Mind/Brain Sciences, University of TrentoRovereto, Italy; ^3^Department of Mathematics, University of BergenBergen, Norway; ^4^CHU Caen, Clinical Research Department, CHU Caen Côte de Nacre, UNICAENCaen, France

**Keywords:** spatial learning, associative learning, landmark processing, overshadowing, mice

## Abstract

Animals use distal and proximal visual cues to accurately navigate in their environment, with the possibility of the occurrence of associative mechanisms such as cue competition as previously reported in honey-bees, rats, birds and humans. In this pilot study, we investigated one of the most common forms of cue competition, namely the overshadowing effect, between visual landmarks during spatial learning in mice. To this end, C57BL/6J × Sv129 mice were given a two-trial place recognition task in a T-maze, based on a novelty free-choice exploration paradigm previously developed to study spatial memory in rodents. As this procedure implies the use of different aspects of the environment to navigate (i.e., mice can perceive from each arm of the maze), we manipulated the distal and proximal visual landmarks during both the acquisition and retrieval phases. Our prospective findings provide a first set of clues in favor of the occurrence of an overshadowing between visual cues during a spatial learning task in mice when both types of cues are of the same modality but at varying distances from the goal. In addition, the observed overshadowing seems to be non-reciprocal, as distal visual cues tend to overshadow the proximal ones when competition occurs, but not vice versa. The results of the present study offer a first insight about the occurrence of associative mechanisms during spatial learning in mice, and may open the way to promising new investigations in this area of research. Furthermore, the methodology used in this study brings a new, useful and easy-to-use tool for the investigation of perceptive, cognitive and/or attentional deficits in rodents.

## Introduction

As postulated by O’Keefe and Nadel (1978), spatial learning is traditionally classified into two types. The first one, called taxon learning, is supported by an association, or a chain of associations, and works by attaching a valence to one or several stimulus(i). Such chains of association between stimuli and a reward notably underlie route-following abilities, classically reported in insects (Collett and Collett, 2002; Collett, 2009). The second type of spatial learning required in a rich environment with multiple stimuli (termed place, or locale learning) is based on the construction of a navigation map that encodes the spatiotemporal relationships between the position of the subject in the environment, its movements and the location of sites of reward. Once the spatial map has been created, it can be updated for a better and finer differentiation of places but no new map will be built despite repeated exposures to the same environment. In that sense, associative mechanisms such as cue competition have for a long-time been assumed to occur in the simplest taxon learning but not in more complex spatial learning when the creation and use of a spatial cognitive map of the environment is required.

Since these pioneering works, some authors have suggested that such a distinction was not so clear. Indeed, the important issue would concern the content of the spatial information encoded in a broadly defined spatial map, rather than how animals learn about space (Gallistel, 1993, 1994; for review see: Bennett, 1996). According to this view, it is now known that animals are able to generate a complex spatial map of their environment (by joining together in a piecemeal fashion several simple spatial representations acquired on the basis of associative learning) by a mechanism called associative integration (Blaisdell and Cook, 2005; Sawa et al., 2005; Chamizo et al., 2006; see also for review: Blaisdell, 2009; Leising and Blaisdell, 2009). In the same line of research, associative mechanisms such as cue competition are likely to also occur during the creation of such a cognitive spatial map: when numerous cues are available in the environment, a weighting has to be carried out by the brain, resulting in more importance given to some cues than to others and/or to an easier use of these cues for learning through associative processes. In agreement with this theory, the associative mechanisms of cue competition have now been found to occur during various spatial tasks in both rats, pigeons or humans (reviewed in: Chamizo, 2002; Prados and Redhead, 2002; Leising and Blaisdell, 2009; but also see Jeffery, 2010).

Cue competition implies a variety of mechanisms that occur during associative learning; the two most common forms of which are overshadowing and blocking (Pavlov, 1927; Kamin, 1969). During Pavlovian conditioning, two stimuli can be in competition for the association with an unconditioned stimulus. Blocking is observed when prior establishment of one element of a compound cue as a signal for reinforcement reduces or blocks the amount learned about a second (for review see: Chamizo, 2002). Overshadowing refers to the finding that the presence of a second relevant cue at the time of learning could potentially cause animals to learn less about a first than they would have done if trained on the first cue in isolation (for review see: Chamizo, 2002).

Transposed to spatial learning, overshadowing means that if a subject has the possibility to use at least two types of cues to initially orientate, the spatial performance may not be equal during subsequent presentation of the cues separately or when cues are placed in conflict in a subsequent test trial (for review, see: Chamizo, 2002). Indeed, the performance can be reduced when both types of cues are presented alone as the result of a reciprocal overshadowing, both cues having competed equally for association (i.e., perceived as equally salient) during the acquisition of spatial knowledge. Alternatively, one type of cue can also overshadow the other one during learning, but not vice versa, an effect named non-reciprocal overshadowing (for review, see: Chamizo, 2002). Several factors, such as the use of an experimental design that will allow balanced initial investigation of all the reinforcers (as well as a similar number of reinforcements) should be taken into careful consideration in studies which focus on the occurrence of cue competition during spatial learning as they have been shown to influence the interpretation of the results. Indeed, in an original study in rats, Diez-Chamizo et al. (1985) provided evidence of overshadowing of intra-maze by extra-maze cues during a spatial learning task in a radial maze but not reciprocal overshadowing of extra-maze by intra-maze cues. However, using a similar apparatus but with a slightly different experimental procedure that controlled for the number of reinforcers (as well as reinforced/unreinforced spatial learning trials using proximal and distal cues) the same group demonstrated subsequently that this overshadowing effect was actually reciprocal (March et al., 1992). Interestingly, Craig et al. (2005) reported that during an object exploration task in rats, proximal cues displacement overshadowed any distal cues displacement. Similarly, an overshadowing of farther landmarks by the ones situated closer to the goal was reported in species of different taxa such as honey-bees (Cheng et al., 1987), birds (European jays: Bennett, 1993; pigeons: Spetch, 1995) and humans (Spetch, 1995). Finally, cue competition can occur between cues from different modalities. For example, reciprocal overshadowing processes have been found to occur between visual and auditory cues during a water-maze task in rats (Sánchez-Moreno et al., 1999), and between tactile intra-maze and visual extra-maze cues during a radial-maze task in rats (March et al., 1992).

From an evolutionary and comparative standpoint, understanding the bases of spatial learning across species is of large interest and this in order to determine similarities but also differences in cognitive abilities and related neuronal and molecular substrates. Thus, although the phenomenon of overshadowing has been the subject of intense research in rats, the results obtained cannot be generalized to mice because of neuroanatomical, neurochemical and behavioral differences between the two species (Routh et al., 2009; Snyder et al., 2009). For example, distinctions of the hippocampal physiology between rats and mice correlate with distinct spatial abilities (Schwegler and Crusiob, 1995; McNamara et al., 1996).

The present study therefore aimed to investigate for the first time in mice the possibility that overshadowing processes can occur between cues during a spatial learning task, when the cues distributed at 360° across the environment are of the same modality, but situated within the immediate environment or farther.

## Materials and Methods

### Subjects and Housing

**Figure 1:**
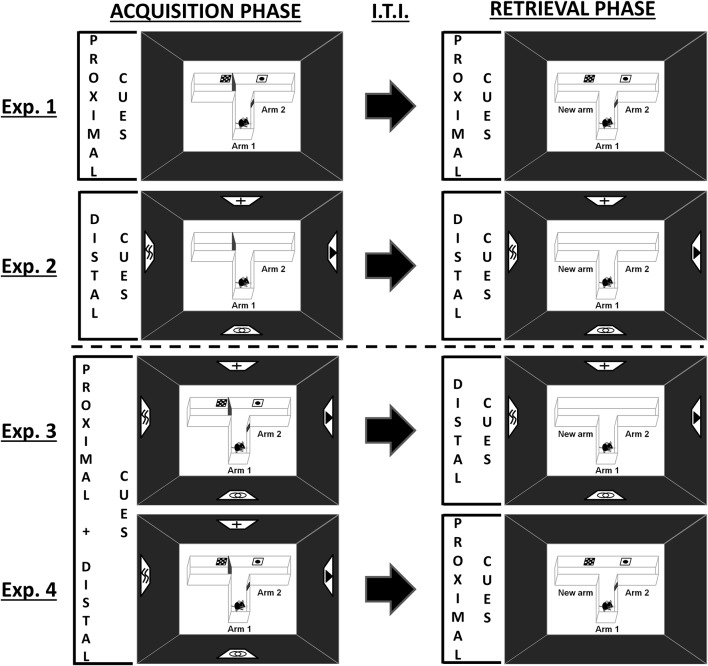
Schematic representation of the behavioral tasks. During the acquisition phase, one arm of the T-maze was randomly closed with a guillotine door. Each mouse was placed a first time in the starting arm (Arm 1) and allowed to visit the two accessible arms (Arm 1 and Arm 2) for 5 min. Mice were then returned to their home cage for $2\frac{1}{2}$ h, before being subjected to the retrieval phase, in which they had free access to each of the three arms for 5 min. Experiments 1 and 2 were designed to investigate spatial performance with proximal or distal visual cues alone, respectively. Experiments 3 and 4 were designed to investigate the existence of an overshadowing of one type of visual cues by the other (Experiment 3: proximal cues removal for the retrieval phase; Experiment 4: distal cues removal for the retrieval phase). Exp.: Experiment; I.T.I.: Intertrial Interval.

Eleven to thirteen weeks old C57BL/6J × Sv129 male mice (*N* = 61), divided in four independent groups (Experiments 1–4; Figure 1), were housed in animal care facilities (Centre Universitaire de Ressources Biologiques (CURB), Caen, France; approval n° A14118015). The mice were maintained in standard polypropylene cages (37 × 23.5 × 18 cm, Charles River, L’Arbresle, France; 5–6 mice per cage) in a temperature and humidity controlled room (12-h light/dark inverted cycle) and had free access to water and food. All the behavioral tests were performed between 8AM–5PM in a pseudo-randomized fashion within each testing day and cage. The principal investigator (VA, personal license number 14-73) was accredited by the Direction Départementale des Services Vétérinaires. The animal investigations were performed under the current European directive (2010/63/EU) as incorporated in national legislation (Decree 87/848) and in authorized laboratories (GIP Cyceron; approval n° E14118001). “Comité d’Ethique NOrmandie en Matière d’EXpérimentation Animale” (CENOMEXA) certifies that this study did not require referral to the regional ethics committee.

### Apparatus and Behavioral Procedure

Mice were given a two-trial place recognition task in a T-maze based on the novelty free-choice exploration paradigm previously developed to study spatial memory processes in rats and mice (Dellu et al., 1992, 2000; Obiang et al., 2011). The standard T-maze used in this study was constructed of white plastic with three identical arms (31 × 8 × 15 cm; BMP Chaudronnerie, Bretteville-sur-Odon, France), and located in a room with dim illumination (<6 lx). Dim illumination was used to allow the mice to see properly all the visual cues as well as the maze details. This procedure, mimicking full moonlight in the wild, is assumed not to induce an exaggerate perturbation of the nocturnal cycle of mice (Kronfeld-Schor et al., 2013; Upham and Hafner, 2013). Before the task and during inter-trial interval, mice were kept in another part of the same testing-room with a similar luminosity in order to prevent stress. We designed a new version of this spatial task in which mice had to build spatial representation of the environment by using specifically proximal and/or distal landmarks (Figure 1; Supplementary Figure S1). Black curtains surrounded the T-maze in order to prevent the interference of non-controlled visual cues. Intra-maze cues designed as proximal cues (posters of 5 × 5 cm; Supplementary Figure S1) were positioned bilaterally on the walls of the maze at the entrance of each arm (4.5 cm from the top and the bottom of the maze). In this way, all cues were visible from the central part of the maze where mice spent a lot of time investigating the environment before choosing a specific arm to explore. Extra-maze distal cues (posters of 20 × 20 cm; Supplementary Figure S1) were suspended on the black curtains of the room walls at a distance of 123 cm from the maze (from the top of the cues to the center of the maze). The behavioral procedure consists of two trials separated by a $2\frac{1}{2}$ h inter-trial interval. During the acquisition phase (trial 1), one arm of the maze was randomly closed with a guillotine door. Each mouse was then placed in the start arm (Arm 1), the head facing away from the center of the maze, and allowed to visit the two accessible arms (Arm 1/Arm 2) for 5 min. At the end of the trial, the labeled mouse was then replaced in its home cage. During the retrieval phase (trial 2), animals had free access to all three arms (Arm 1/Arm 2/New arm) for 5 min. Given that duration of exploration is an index of inspective exploration (i.e., the exploration of particular items), whereas number of explorations corresponds to inquisitive exploration (i.e., the active exploration of the environment as a whole), this last parameter was chosen to assess the behavioral performance of the mouse in the maze (Berlyne, 1960; Łukaszewska and Młodkowska, 1980; Dellu et al., 2000). Thus, spatial memory was assessed through the comparison of the number of visits in each arm (considered only when the mouse passed two-thirds of the arm) during the 5 min of the retrieval phase. At the end of each 5-min phase, the apparatus was entirely cleaned with alcohol in order to eliminate chemical cues, and dried with absorbent tissue.

Experiments 1 and 2 were designed to investigate spatial performance with proximal or distal visual cues alone (Figure 1, single-cue condition). As such, proximal (Experiment 1) or distal (Experiment 2) cues were presented all along the two phases of the learning task. Experiments 3 and 4 tested whether an overshadowing occurs between the proximal and distal visual cues (Figure 1). Thus, the two types of cues were available during the acquisition phase (compound-cue condition), but one of them was removed during the retrieval phase (Experiment 3: proximal cues removal; Experiment 4: distal cues removal).

### Data Analysis and Statistics

Statistical tests and boxplots were performed using the R software (version 3.1.2). Analysis of the data sets via parametric approaches, e.g., generalized linear (mixed effects) models turned out to be inappropriate in the majority of cases due to violation of residual normality (Shapiro-Wilk’s tests). Therefore, we chose a non-parametric approach for all analyses. Friedman’s tests were used in order to investigate effects of factors with three levels in a repeated measures setting. When an appropriate significant effect was detected by Friedman’s tests, we performed *post hoc* analysis using Wilcoxon’s signed-rank tests for matched samples (Siegel and Castellan, 1988), with *p-values* adjusted by Holm’s method in order to account for multiple comparisons (Holm, 1979). Additionally, we performed multifactorial “F1-LD-F1” non-parametric analyses of variance (“nparLD” R package) between “Single-Cue” and “Compound-Cue” experiments. This non-parametric analysis technique is particularly suitable for data with repeated measures in factorial experiments and served conjointly for examination of the effects and possible interactions of both the experimental procedures (cues available during the training phase) and repeated factors (pattern of visits to the arms; Brunner et al., 2002; Noguchi et al., 2012). An alpha level of *p* < 0.05 was used for determination of significance in all statistical tests; all tests are two-tailed.

## Results

### Mice Successfully use Both Distal and Proximal Visual Cues Presented Separately to Navigate

**Figure 2:**
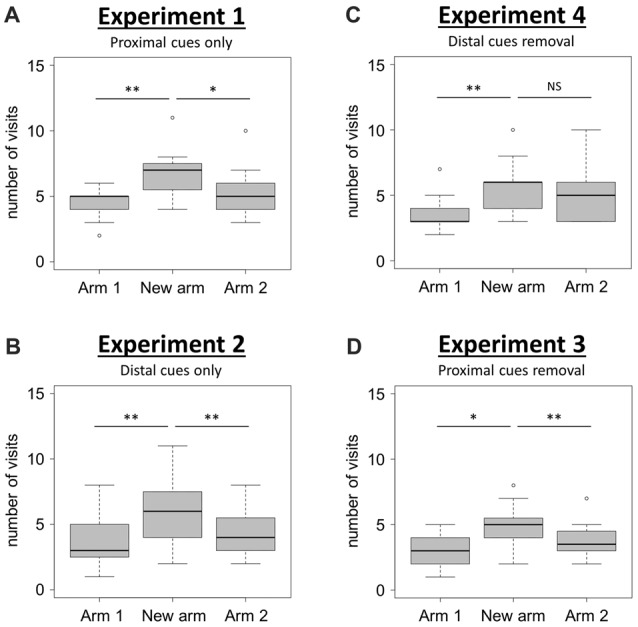
Spatial performance in the T-maze. **(A,B)** Spatial performance when proximal (Experiment 1; **A**), or distal (Experiment 2; **B**), visual cues were available during both the acquisition and the retrieval phases. **(C,D)** Spatial performance when both proximal and distal visual cues were available during the acquisition phase, but one type was removed for the retrieval phase (Experiment 4, **C**: distal cues removal; Experiment 3, **D**: proximal cues removal). Spatial performance was assessed through the comparison of the number of visits in each of the three arms during the retrieval phase. Friedman tests: *P* < 0.01 for all tests. Wilcoxon signed-rank tests: *P < 0.05; **P < 0.01; NS: *P* > 0.05. *N*_Experiment 1_ = 15; *N*_Experiment 2_ = 15; *N*_Experiment 3_ = 16; *N*_Experiment 4_ = 15. Boxplots show distributions with black horizontal lines indicating the median, box margins denoting the lower and upper quartiles, and the whiskers extending from the box out to the most extreme observation within 1.5 times the interquartile range from the box.

When using only proximal cues (Experiment 1), mice visited the new arm significantly more often than the two familiar arms (Arms 1 and 2) of the T-maze (Figure 2A; Friedman test: *χ*^2^ = 13.45, *df* = 2, *N* = 15, *P* = 0.001; Wilcoxon signed-rank tests: “New arm vs. Arm 1”, *V* = 3.5, *P* = 0.004, “New arm vs. Arm 2”, *V* = 74, *P* = 0.048). This result shows that mice are able to discriminate between the newly opened arm and the two familiar arms during the retrieval phase, therefore indicating good spatial memory performance when solely using proximal visual cues.

When using only distal cues (Experiment 2), mice also exhibited a good spatial memory performance since they correctly discriminated between the newly opened arm and the two familiar arms (Figure 2B; Friedman test: *χ*^2^ = 12.44, *df* = 2, *N* = 15, *P* = 0.002; Wilcoxon signed-rank tests: “New arm vs. Arm 1”, *V* = 2.5, *P* = 0.006, “New arm vs. Arm 2”, *V* = 8.5, *P* = 0.006). These two first experiments therefore show that mice are capable to use either proximal or distal visual cues alone to accurately navigate in the T-maze. The question of the occurrence of an overshadowing effect (i.e., a different salience given by the brain to proximal and distal visual cues when both of them are available during the acquisition phase), was therefore examined in Experiments 3 and 4.

### Distal Visual Cues Can Overshadow the Proximal Ones during Spatial Learning, but Not Vice Versa

When proximal cues were removed for the retrieval phase (Experiment 3), mice discriminated correctly the newly opened arm from the others since they visited significantly more often the new arm than arms 1 and 2 (Figure 2D; Friedman test: *χ*^2^ = 11.21, *df* = 2, *N* = 16, *P* = 0.004; Wilcoxon signed-rank tests: “New arm vs. Arm 1”, *V* = 12, *P* = 0.013, “New arm vs. Arm 2”, *V* = 105.5, *P* = 0.008). During this condition, they performed the task successfully, therefore indicating that proximal cues had not overshadowed the distal ones.

By contrast, when distal cues were removed during the retrieval phase (Experiment 4), mice failed to discriminate one familiar arm (Arm 2) from the newly opened arm (Figure 2C; Friedman test: *χ*^2^ = 11.16, *df* = 2, *N* = 15, *P* = 0.004; Wilcoxon signed-rank tests: “New arm vs. Arm 1”, *V* = 2, *P* = 0.005, “New arm vs. Arm 2”, *V* = 45.5, *P* = 0.636). This observation reveals that the removal of distal visual cues during the retrieval phase alters the ability of mice to orientate properly. This result points towards the occurrence of a non-reciprocal overshadowing effect of proximal by distal visual cues during the presently reported spatial task in mice.

We then compared each “Compound-Cue” condition (Experiments 3 and 4) to its respective “Single-Cue” control condition (Experiments 2 and 1, respectively). The rationale was to further validate the occurrence of this overshadowing phenomenon by showing that removal of the distal visual cues in Experiment 4 led to abnormal spatial performance when compared to the condition with only proximal visual cues through the whole experiment (Experiment 1). As hypothesized, mice tested in Experiments 2 (acquisition and retrieval phases with distal cues alone) and 3 (acquisition phase with both proximal and distal cues; retrieval phase with only distal cues) displayed similar performance during the retrieval phase, with only an “Arms” effect detected (“F1-LD-F1” non-parametric analyses of variance; Wald-type test; “Arms”: *χ*^2^ = 29.03, *df* = 2, *P* = 4.973e-7; “Experiments”: *χ*^2^ = 1.50, *df* = 1, *P* = 0.221; “Arms*Experiments”: *χ*^2^ = 0.449, *df* = 2, *P* = 0.799). Despite observing a strong “Arms” and a residual “Groups” effects, we failed to detect an interaction between these two factors when comparing Experiments 1 (acquisition and retrieval phases with proximal cues alone) and 4 (acquisition phase with both proximal and distal cues; retrieval phase with only proximal cues; “F1-LD-F1” non-parametric analyses of variance; Wald-type test; “Arms”: *χ*^2^ = 47.10, *df* = 2, *P* = 5.919e-11; “Experiments”: *χ*^2^ = 3.91, *df* = 1, *P* = 0.048; “Arms*Experiments”: *χ*^2^ = 1.48, *df* = 2, *P* = 0.476). It is noteworthy that, similar results were obtained when comparing the two “Single-Cue” experiments (Experiments 1 and 2; “F1-LD-F1” non-parametric analyses of variance; Wald-type test; “Arms”: *χ*^2^ = 33.30, *df* = 2, *P* = 5.866e-8; “Experiments”: *χ*^2^ = 1.51, *df* = 1, *P* = 0.189; “Arms*Experiments”: *χ*^2^ = 0.524, *df* = 2, *P* = 0.769) but also when comparing the two “Compound-Cue” experiments (Experiments 3 and 4; “F1-LD-F1” non-parametric analyses of variance; Wald-type test; “Arms”: *χ*^2^ = 38.10, *df* = 2, *P* = 5.339e-9; “Experiments”: *χ*^2^ = 7.993, *df* = 1, *P* = 0.08; “Arms*Experiments”: *χ*^2^ = 1.18, *df* = 2, *P* = 0.554).

## Discussion

This study presents the first prospective assessment of the existence of an overshadowing effect, one form of cue competition, between visual landmarks during spatial learning in mice. When both types of cues are simultaneously available in the environment, distal information rather than the proximal one tends to be preferentially encoded in the mouse brain for a proper subsequent navigation. These findings reported for the first time in mice, albeit obtained on a small population, may be explained by the central role of the associative processes that occur during spatial learning.

Several studies have shown that animals are able to generate, through an inference-like process, a complex spatial map of their environment based on the integration of different sets of spatial relationships acquired with associative processes (Blaisdell and Cook, 2005; Sawa et al., 2005; Chamizo et al., 2006; reviewed in: Prados and Redhead, 2002; Blaisdell, 2009). By using this process, animals can compute relationships between any targeted spatial location and landmarks, even if no direct association occurred before between the goal and one particular landmark. Applied to the present study, this means that during the acquisition phase, mice were able to generate a complex spatial map based on several simple associations made between the different locations and the distal or proximal visual cues presented alone. Based on this previously generated spatial map, mice were therefore able to recognize the “New Arm” as a new place when they were again placed in the maze for the retrieval phase (Experiments 1 and 2). This also holds true when both cues were presented as a compound during the acquisition phase followed by the removal of proximal cues for the retrieval phase (Experiment 3) but not when the distal visual cues were removed for the retrieval phase (Experiment 4). A couple of hypotheses can be raised in an attempt to explain the overshadowing phenomenon observed in this set of experiments. First, a deficit of learning about proximal cues could have occurred when both of the visual landmarks were available during the acquisition phase. Some behavioral evidence from rodents suggests that novelty serves as a positive reinforcer, such that rodents will explore novel places when they are allowed to choose freely among unknown and familiar places (Pierce et al., 1990; Klebaur and Bardo, 1999; Deacon and Rawlins, 2006). This innate preference for unfamiliar environments can be explained by the rewarding effect of spatial novelty. Indeed, it has been evidenced for example that the exploration of a new environment induces the activation of the mesolimbic dopaminergic system in the same way as drug abuse (Bardo et al., 1996; Rebec et al., 1997; Klebaur and Bardo, 1999; Hughes, 2007). We can therefore infer that the increase of visits in the newly opened arm of the maze during the retrieval phase of the Single-Cue conditions (Experiments 1 and 2) is induced by spatial novelty predicted by distal, or proximal, visual cues. Moreover, these data confirm the ability of mice to acquire and retain spatial knowledge in such a paradigm (Dellu et al., 1992, 2000). The navigational deficits observed when distal cues (Experiment 4), but not proximal cues (Experiment 3), were removed for the retrieval phase of the Compound-Cue condition suggest that the proximal cues-associated reward value of spatial novelty, which normally motivates spatial behavior, was reduced. One can thus hypothesize that a competition between the two types of landmarks has occurred during the acquisition phase, in such a way that the distal cues were given more weight and attention. In other words, the proximal visual cues might have not enough reward value when presented as a compound with the distal ones, so they have been relatively ignored during the first trial, leading to an overshadowing of intra-mazes cues by extra-mazes cues. However, an alternative explanation can be that the attention paid to both sets of cues was similar during the acquisition phase, but that the recall performance was altered due to the perception of the whole maze as a new space. Indeed, Julian et al. (2015) have shown that mice are able to use the context of the environment represented by proximal visual landmarks in the arena, to recognize places. One could therefore hypothesize that the removal of the entire set of distal cues for the retrieval phase in Experiment 4 led mice to identify the maze as a new environment, with all locations treated as new and worth exploring. These hypotheses will have to be investigated in more detail in future studies.

In this study, an overshadowing phenomenon was observed in the Compound-Cue condition where the proximal cues were less used than the distal ones. Analyses of variance failed to detect any positive “Arms*Experiments” interactions between Experiments 1–4 and 2–3. At first sight, these results would suggest that, even if a specific pattern was identified in Experiment 4 (the mice were unable to discriminate the new arm after the removal of the distal cues for the retrieval phase), the overall pattern of response did not differ from the other experiments. However, before ruling out the possibility that an overshadowing effect occurred in the present experiments, we would like to focus on two main explanations that may have led to this overall absence of interaction effect. Firstly, as proposed by the developers of the two-trial place recognition task have shown, the best way to analyze spatial performance is to perform intragroup comparisons of the visits (for inquisitive behavior), or the duration (for study of inspective behavior) into the new arm compared to the familiar arms during the retrieval phase of the learning task (Dellu et al., 1992, 2000). Therefore, in order to efficiently perform multifactorial analyses, the results could be duplicated using behavioral tools adapted to the detection of intergroup effects such as the Morris water-maze or the radial-maze. Second, the two-trial place recognition procedure was mainly used to assess genetic variability as well as cellular and molecular bases of spatial learning in rodents (Dellu et al., 1992, 2000; Obiang et al., 2011, 2012; Hébert et al., 2016). The effects reported in these publications can therefore reasonably be assumed to be far larger than the one observed in our study, where some fine difference in behavior has to be assessed between otherwise similar healthy mice. Thus, according to power analyses run on the data obtained in the present study, a strong increase of the number of experimental subjects would, in probability, have achieved the expected effect: “Arms*Experiences” interactions.

One could object that, with the T-shaped maze used in this study, mice would have used the geometric features provided by the apparatus rather than the available visual cues to orientate properly (for review about the use of geometrical features during spatial learning, see: Cheng and Newcombe, 2005). Although we cannot rule out a possible contribution of geometrical cues in the present experiments, it should be mentioned that such a distinction between the use of geometric and allothetic information during spatial navigation is not clearly understood. Some authors have for example reported an influence of the geometric configuration of environmental landmarks on spatial navigation in rats (Greene and Cook, 1997; Benhamou and Poucet, 1998). It is also well known that geometrical features are needed to scale entorhinal grid cell firing, thereby influencing the hippocampal place cells whose firing is mediated by environmental visual cues (Shapiro et al., 1997; Renaudineau et al., 2007; Scaplen et al., 2014). The relative contribution of the different set of cues used in our study as well as the role played by the geometrical features of the maze should be addressed with more details in future studies, using for example a Y-shaped maze presenting less conspicuous geometrical features than the T-maze.

To finish, a research group has previously identified the existence of two landmark-processing systems for distal and proximal visual cues during a spatial task, the two converging to the hippocampus (Save and Poucet, 2000; Parron et al., 2004). Whereas the one devoted to the processing of distal cues is dependent on the entorhinal cortex (EC) *per se*, the one devoted to the processing of proximal cues requires the activation of the associative parietal cortex (APC). We recently extended these results by showing that the encoding of proximal vs. distal landmarks is mediated not only by different anatomical pathways, but also by different molecular mechanisms with tissue-type plasminogen activator (tPA)-dependent N-methyl-D-aspartate receptor signaling pathway in the EC playing a key role in distal but not proximal landmark processing (Hébert et al., 2016). In this same study, we have also provided the first evidence of the occurrence of a negative tone exerted by tPA-related EC/hippocampus-dependent pathway on the APC/hippocampus-dependent pathway. This kind of negative tone, recently mentioned in other contexts (Winocur et al., 2013), would ultimately act by giving an exaggerated salience to the distal cues, therefore preventing the use of the proximal ones. The overshadowing effect of proximal by distal cues identified in our present study would therefore be in agreement with the above reports. Naturally, additional investigations will be needed to confirm these results in other spatial navigation tasks in mice, as well as for the identification of the molecular and cellular mechanisms underlying this process.

To the best of our knowledge, this is the first study focusing on cue competition during a spatial learning task in mice. Our prospective findings provide a first set of clues in favor of the occurrence of an overshadowing of one category of cues (distal cues) on the other one (proximal cues) of a same (visual) modality during a spatial task in mice. In addition, this pilot study provides a new behavioral paradigm that might be useful not only to investigate the occurrence of associative mechanisms during spatial learning but also to characterize the phenotype of mice models extensively used in genetic and behavioral researches about perceptive, cognitive and/or attentional pathologies.

## Author Contributions

MH, JB, DV and VA designed the study; analyzed the data; wrote and edited the manuscript; approved the final version of the manuscript. MH performed the experiments.

## Conflict of Interest Statement

The authors declare that the research was conducted in the absence of any commercial or financial relationships that could be construed as a potential conflict of interest.

## Supplementary Material

FIGURE S1 Representation of visual cues used in the study. **(A)** Distal visual cues consisted of four white posters (20 × 20 cm) displaying different black geometrical shapes. They were suspended on the black curtains of the room walls at a distance of 123 cm from maze (from the top of the cues to the center of the maze). **(B)** Proximal visual cues consisted of three sets of white posters (5 × 5 cm) displaying contrasted black motifs. Each set was fixed bilaterally on the walls at the entrance of each arm (4.5 cm from the top and the bottom of the maze). The arrangement of visual stimuli (distal and/or proximal) was maintained constant for all mice across the same experiment, but modulated as much as needed according to the experimental design (i.e., removal of specific cues for the retrieval phase in Experiments 3 and 4).
