# Supplementary figures and images for: Are Distal and Proximal Visual Cues Equally Important during Spatial Learning in Mice? A Pilot Study of Overshadowing in the Spatial Domain
Source: Front Behav Neurosci. 2017 Jun 6;11:109. doi: 10.3389/fnbeh.2017.00109 (PMC5459897; doi:10.3389/fnbeh.2017.00109)

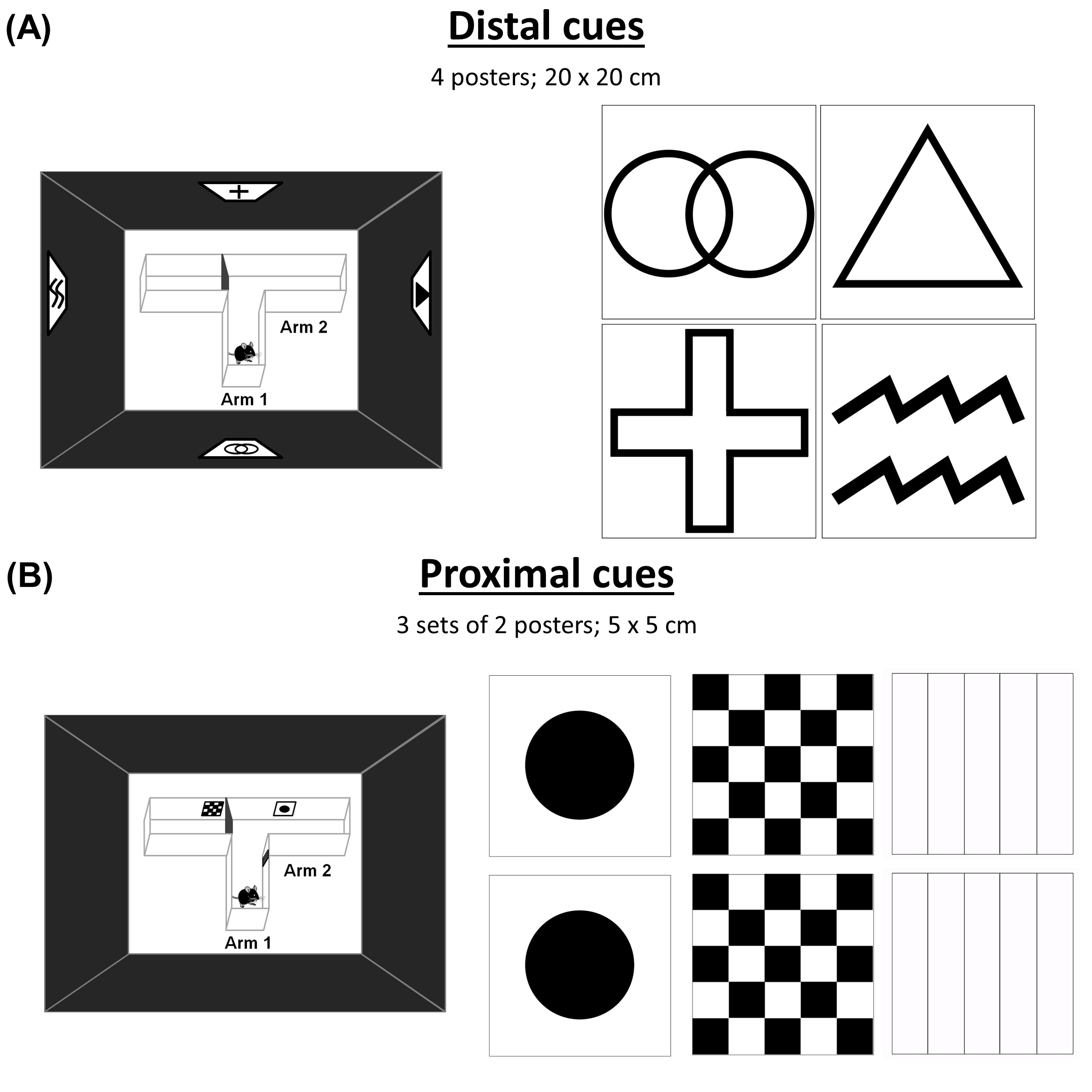

Supplement: FIGURE S1 — Representation of visual cues used in the study. (A) Distal visual cues consisted of four white posters (20 × 20 cm) displaying different black geometrical shapes. They were suspended on the black curtains of the room walls at a distance of 123 cm from maze (from the top of the cues to the center of the maze). (B) Proximal visual cues consisted of three sets of white posters (5 × 5 cm) displaying contrasted black motifs. Each set was fixed bilaterally on the walls at the entrance of each arm (4.5 cm from the top and the bottom of the maze). The arrangement of visual stimuli (distal and/or proximal) was maintained constant for all mice across the same experiment, but modulated as much as needed according to the experimental design (i.e., removal of specific cues for the retrieval phase in Experiments 3 and 4). [file Image_1.tif]
